# Comparison Between Crystalline and Amorphous Silicon as Anodes for Lithium Ion Batteries: Electrochemical Performance from Practical Cells and Lithiation Behavior from Molecular Dynamics Simulations

**DOI:** 10.3390/ma18030515

**Published:** 2025-01-23

**Authors:** Geonhee Kim, Min-Ji Yang, Sanghun Lee, Jae-Hyun Shim

**Affiliations:** 1 Department of Chemistry, Gachon University, Seongnam 13120, Republic of Korea; geonheekim@gachon.ac.kr; 2 Department of Energy System Engineering, Dongshin University, Naju 58245, Republic of Korea; alsals6847@dsu.ac.kr

**Keywords:** lithium ion battery, anode materials, crystalline silicon, amorphous silicon, molecular dynamics, lithiation

## Abstract

As a prominent next-generation anode material for high-capacity applications, silicon stands out due to its potential. Crystalline silicon, which offers a higher initial capacity compared to its amorphous counterpart, presents challenges in practical applications due to its poor cycling performance. In this study, we prepared composites of crystalline and amorphous silicon with graphite, assembled pouch-type full cells, and evaluated their suitability for practical use. The material incorporating amorphous silicon demonstrated superior performance at both high and low rates, as well as various temperatures. Additionally, the changes in cell thickness during charge and discharge, i.e., the volume changes in the anode material, are significantly related to cycling performance. We examined the microscopic interactions between silicon and lithium atoms using molecular dynamics simulations. Our observations indicate that lithium migration within amorphous silicon, which has lower activation energy, is much easier than in crystalline silicon. In crystalline silicon, lithium penetration is greatly influenced by the orientation of the crystal planes, resulting in anisotropic volume expansion during lithiation.

## 1. Introduction

Lithium ion secondary batteries (LIBs), long relied upon as energy storage systems for portable electronic devices, are now rapidly gaining ground in the electric vehicle market. However, for electric vehicles to win a leading position in the automotive industry, LIBs designed for high mileage must overcome significant challenges, with increasing their capacity being the most critical of them.

Since the commercialization of LIBs in the early 1990s, graphite has stood out as the most balanced anode material in terms of various battery performance factors, such as capacity, power, safety, and cost. However, with the capacity of graphite (theoretical capacity of 372 mAh g^−1^) being adequate for small electronic devices but insufficient for the high demands of electric vehicles, researchers have started exploring alternative anode materials to replace graphite for application in next-generation LIBs. Materials that undergo electrochemical cycling via an alloying/dealloying mechanism are likely to exhibit higher capacities than graphite, which operates through an intercalation/deintercalation mechanism. After confirming that various metal elements can form alloys with lithium through an electrochemical process [1], different compositions of Li-Si alloys were identified during reversible charge–discharge cycling [2]. Li_22_Si_5_, which boasts the highest ratio of Li to Si among the reported empirical formulas, is recognized for its theoretical capacity of ~4200 mAh g^−1^. Silicon’s lithiation mechanism, which offers the inherent advantage of having a high capacity as an anode material, also features the inevitable disadvantage of the significant volume difference between its charged and discharged states. This drawback, when combined with repeated charging and discharging, leads to mechanical deterioration of the silicon anode material, resulting in pulverization. The significantly increased surface area of the pulverized materials contributes to serious side reactions and a loss of electrical contacts. Consequently, LIBs using silicon anodes experience huge irreversible capacity with rapid degradation in cycling due to the consumption of a large amount of lithium ions.

Numerous researchers have dedicated their efforts to addressing the aforementioned issues with silicon and improving its practical viability. Introducing nanostructures of silicon with a stress-relief buffer matrix is the most common approach to accommodating the volume change by increasing the surface area and, consequently, enhancing the binding energy. The simplest idea is making composites of very small (0D) silicon particles with carbon or other materials. The composites containing tens of wt% Si have demonstrated a notable increase in capacity while maintaining a certain level of cyclability [3,4,5,6,7,8,9,10,11,12]. As the technology for manipulating nanostructures has advanced, it has led to the introduction of silicon nanowires [13,14,15,16,17] or nanotubes [18,19,20], which have 1D structures, as well as the development of silicon nanosheets [21,22,23] with 2D structures and porous silicon nanoparticles [24,25,26,27,28] with 3D structures. These advancements have opened up new possibilities for their use as anode materials for LIBs. Several review articles have extensively covered the synthesis, characterization method, structural features, and electrochemical performance of various types of silicon materials [29,30,31,32,33,34]. However, in order to achieve a balanced performance suitable for commercial LIBs, simply increasing the silicon content is not an answer; instead, silicon must be combined with a significantly larger amount of graphite or integrated into composite materials containing graphite to meet the necessary cycling ability. Currently, development has progressed to the stage of 10–20 wt% silicon to ensure practical applicability [35,36,37].

To control the volume expansion of silicon occurring during cycling, it is necessary to understand the lithiation mechanism. The lithiation process of crystalline silicon (c-Si) is commonly known to follow a two-phase mechanism, which refers to a sharp change in lithium concentration at the phase boundary [38,39]. Additionally, the lithiation of c-Si leads to a volume expansion that is highly anisotropic. Specifically, the expansion along the <110> direction is significantly larger than along the <100> or <111> directions. As a result, the fractures in c-Si anodes tend to occur preferentially between neighboring (100) planes [40,41]. This mechanism explains the phenomenon of inhomogeneous volume expansion, leading to fragmentation due to high stress when lithiation occurs [41,42,43]. To avoid the large stress caused by the anisotropic lithiation of c-Si, considerable effort has been directed towards employing amorphous silicon (a-Si) as an alternative. Initially, it was thought that the concentration of Li would gradually change through diffusion, following a one-phase mechanism. However, multiple experimental studies have revealed that the lithiation of a-Si occurs in two distinct steps: first, the sharp phase boundary between a-Si and amorphous Li_x_Si shifts; then, in the second step, no visible interface is observed [44]. In later research, it has been discovered that this two-step process with a two-phase mechanism is only observed during the initial lithiation, and, in subsequent cycles, it transitions into a one-step process occurring in a single phase [45,46]. Therefore, it has been considered that a-Si may have more favorable kinetics and fracture behavior upon lithiation than c-Si, which could make a-Si a more desirable material for certain applications where these properties are important [44,45].

This study compares the electrochemical performance of amorphous and crystalline Si composites with graphite as an anode material for LIBs. Characteristics such as capacity, rate capability, and cycling properties are evaluated. Overall, a-Si demonstrates superior performance in certain aspects, while the crystalline counterpart shows better characteristics in others. Furthermore, molecular dynamics computer simulations are conducted to offer an atomistic description of the lithiation processes in both c-Si and a-Si. The kinetics of lithiation in the two systems exhibit clear differences, and distinct behaviors are observed depending on the orientation of the crystal planes.

## 2. Experimental and Computational Details

This study investigates the electrochemical properties based on the structural characteristics of c-Si (particle size ≤ 100 nm, Nanografi Nano Technology, Boston, MA, USA) and a-Si powders. Specifically, a-Si powders were manufactured using RF (5 kW) plasma pyrolysis synthesis. For this synthesis, SiH_4_ (impurity 99.9999%, flow rate 0.03 L/min) was used as the source gas, being carried by the H_2_ flow (impurity, 99.9999%, flow rate 0.05 L/min). In addition, Ar (impurity 99.9999%, flow rate 2 L/min) was used as the sheath and quenching gas. The a-Si powders were synthesized from the mixture of SiH_4_ and H_2_ (30:70 mol%) at 700 °C for 1 h and 30 min. The decomposed a-Si powders exiting the reactor were collected using a collection device. X-ray powder diffraction patterns were obtained using a Philips X PERT PRO diffractometer (Malvern Panalytical, Malvern, UK) (Cu Kα radiation), equipped with a graphite monochromator and a PIXCel solid-state detector (Malvern Panalytical, Malvern, UK), operating in continuous scan mode at 40 kV and 40 mA. Diffraction patterns were scanned from 2θ = 10° to 60°, with a step size of 0.013° and a scan rate of 0.67°/min. The morphologies of the samples were observed via a field-emission-scanning electron microscope (FT-SEM) (JSM-7500F, JEOL Ltd., Tokyo, Japan) serviced by the Center for Bionano Materials Research at Gachon University (Seongnam, Republic of Korea). Distribution of the crystalline features was also observed under a high-resolution transmission electron microscope (JEM-2100F, JEOL Ltd., Japan) at 200 kV acceleration voltage for crystal structure determination and selected area electron diffraction (SAED) analysis.

In this study, the electrochemical properties of Si-based electrodes were investigated using pouch-type full cells, as detailed in Table S1 (Supporting Information). The anode was prepared by mixing graphite and silicon in a 95:5 weight ratio, while the cathode comprised a commercialized LiNi_0.8_Co_0.1_Mn_0.1_O_2_ material (POSCO Future M Co., Ltd., Pohang, Republic of Korea). An anode plate (3.2 cm × 4.2 cm) and a cathode plate (3.0 cm × 4.0 cm) were assembled in a layered structure with a single PP|PE|PP separator. Then, approximately 3 mL of electrolyte was added to each cell. The electrolyte composition was 1.2 M LiPF6 in a mixture of ethylene carbonate, ethyl methyl carbonate, and dimethyl carbonate (3:4:3 by volume) with additives, including 1.0% fluoroethylene carbonate, 0.5% vinylene carbonate, 2.0% 1,3-propane sultone, 0.2% LiFSI, 0.2% LiBF4, 1.0% LiPO2F2, and 0.5% succinonitrile. To improve experimental accuracy by managing gas evolution during cell formation, the pouch cells were clamped using acrylic plexiglass sheets.

Electrochemical performance tests were conducted at a voltage range of 2.75−4.2 V at room temperature using a battery cycler (BCS-805 Battery Cycling System, BioLogic, Seyssinet-Pariset, France). The cyclability evaluation was performed over 200 cycles, with charging at 0.2 C and discharging at 0.33 C and 1.0 C, respectively. To investigate the temperature effect, these evaluations were carried out at 25 °C and 45 °C. Electrochemical impedance spectra were obtained using an electrochemical workstation (VSP 300, BioLogic, France), with an AC perturbation signal amplitude of 10 mV and a frequency range from 1 MHz to 5 mHz. The electrochemical impedance spectroscopy (EIS) measurements were taken after the first charge at 0.1 C before the electrode was equilibrated and the impedance data were analyzed using EC-Lab software (Version 11.52, BioLogic, Seyssinet-Pariset, France). Swelling measurements during charging and discharging were performed using an in situ thickness measurement device with a pressure resolution of ±0.01 kg and a thickness detection resolution/precision of 0.01 μm/±0.1 μm.

Lithiation of c-Si and a-Si were investigated by molecular dynamics simulation with a Reactive Force Field (ReaxFF) using the LAMMPS package (Version 23, JUN, 2022) [47]. ReaxFF is very suitable for simulating the lithiation process into silicon because it can describe the formation and breaking of bonds between atoms [48]. In this study, we used the ReaxFF developed by Jung et al. to simulate the lithiation of silicon nanowires [49]. This parameter set has been recently validated again by successfully modeling a capacity loss, which is attributed to internal defects within silicon particles, after the first cycling [50]. Although there are several recently published versions of ReaxFF potentials [51,52], there were no significant differences in describing the behavior of Li penetrating through bulk Si atoms. Given that our results reproduce those of previous studies well, we believe that the choice of force field is appropriate [49].

**Figure 1:**
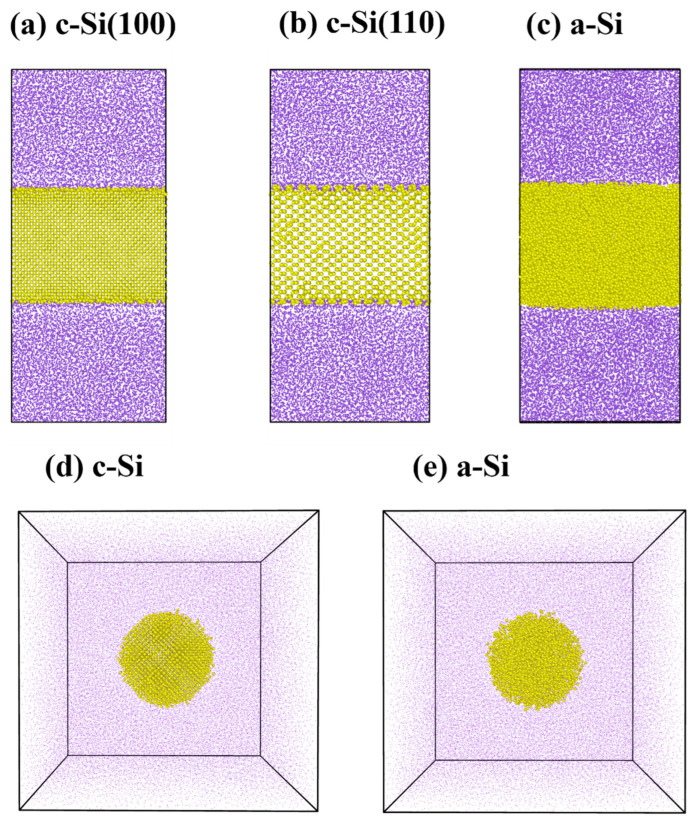
Initial structures for lithiation processes: (**a**) c-Si (100), (**b**) c-Si (110), and (**c**) a-Si nanofilms and (**d**) c-Si and (**e**) a-Si nanospheres. Detailed specifications of the systems are listed in Table 1. (Purple: Lithium, Yellow: Silicon).

In this study, we simulated the lithiation of c-Si and a-Si nano films as well as spherical nanoparticles. For the c-Si (Fd-3m, a = 5.44 Å) system, we constructed two films (total number of Si atoms: 12,506) with a thickness of 5 nm that were oriented along the *z*-axis, as in [100] and [110] directions, in the center of a rectangular parallelepiped box. Then, the remaining empty space was filled with lithium atoms (total number of Li atoms: 20,630), as shown in Figure 1a,b. Meanwhile, the a-Si film was created following the procedure below. A c-Si film with a thickness of 5 nm was positioned in the center of the simulation box. Initially, the temperature was raised to 3000 K at a rate of 27 K/ps over 100 ps to melt the structure. After annealing at 3000 K for 100 ps, the system was cooled down to 300 K at the same rate to form the a-Si structure. After forming a-Si, 21,883 lithium atoms were randomly added to the upper and lower regions. Then, a small number of lithium atoms was additionally added to ensure the initial stress was set to zero. The initial velocities of all atoms were assigned at 300 K, and a minimization process was conducted to maintain equilibrium between the lithium and silicon, continuing until the energy tolerance was less than 10^−3^ or the force tolerance was below 10^−5^ kcal/mol·Å. While some lithiation and diffusion occurred at the silicon–lithium interface, the diffusion distance was negligible (2–3 Å) (Figure 1c).

To ensure that the generated a-Si was sufficiently equilibrated, we analyzed the coordination number (CN) and bond length. In the initial state of a-Si before lithiation, while a small proportion of CN 3 and 5 was observed compared to c-Si, where CN 3 and 5 are almost absent, the majority of the CNs were 4. Additionally, the bond length distribution for Si-Si in a-Si, where dangling bonds are present, had a maximum distribution ~0.1 Å longer than that of c-Si, which is consistent with previous studies [53,54]. These results are presented in Figure S4 of Supporting Information.

For the c-Si spherical nanoparticle system, we placed a crystalline Si particle with a radius of 25 Å in a cubic box with a side length of 130 Å and filled the remaining space with 97,013 lithium atoms (Figure 1d). For the a-Si spherical nanoparticle system, we constructed it in a similar manner with the same number of atoms as in the c-Si spherical nanoparticle system (Figure 1e). The detailed specifications of the systems are listed in Table 1.

We conducted the NVT (constant number of particles, volume, and temperature) MD simulations at 300 K using a Nosé–Hoover thermostat [55,56], applying periodic boundary conditions in all directions. The simulations for the films and the spheres were conducted for 500 ps and 50 ps, respectively, with the time step of 0.5 fs. For the charge calculation between the atoms, the charge equilibration approach [57] was employed every step within the cut-off radius of 10 Å. Prior to the simulation, a simple energy minimization process was performed to optimize the system and prevent unrealistic atomic movements resulting from excessively large forces acting on atoms in too close a proximity in the initial state. Additionally, to set the initial stresses to zero, the z-dimension of the simulation box was finely adjusted. The formation of the Si-Li alloy on the Si surface during the minimization process was so minor that it can be ignored.

## 3. Results and Discussion

### 3.1. Structural Analysis of Silicon Particles

**Figure 2:**
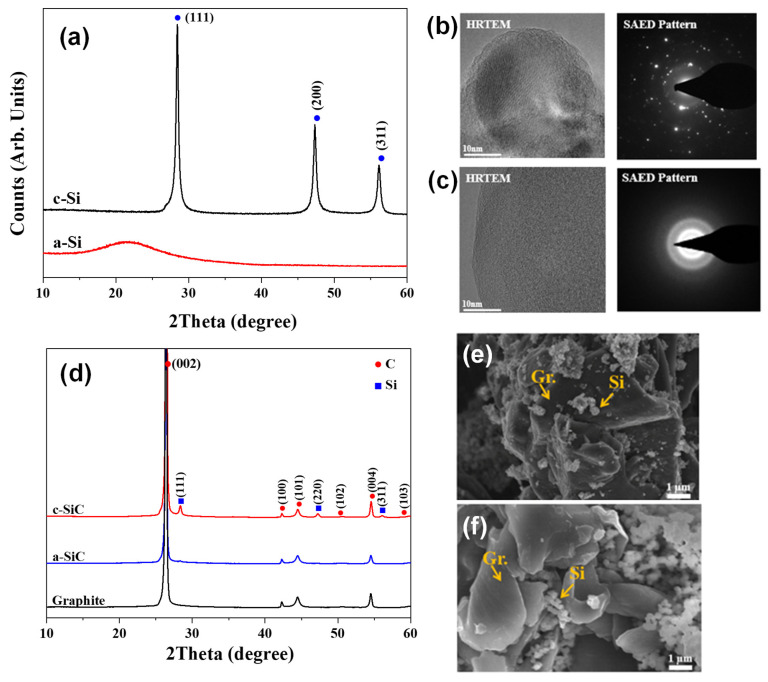
Characterization of materials: (**a**) XRD patterns of c-Si and a-Si; HRTEM images with a SAED pattern of (**b**) c-Si and (**c**) a-Si; (**d**) XRD patterns of c-SiC and a-SiC; and an SEM image of (**e**) c-Si and (**f**) a-Si.

Figure 2a shows the XRD data of c-Si and a-Si, while Figure 2b,c display high-resolution transmission electron microscopy (HRTEM) images with SAED patterns of c-Si and a-Si, respectively. In the XRD pattern of c-Si, distinct diffraction peaks corresponding to the (111), (220), and (311) crystal planes are evident, consistent with the cubic Fd-3m structure observed in its SAED pattern. Conversely, a-Si, synthesized through plasma thermal decomposition, exhibits a purely amorphous structure, with no nano-scale crystalline phase visible in either the XRD pattern or the HRTEM image with the SAED pattern. Figure 2d presents XRD data obtained after blending c-Si and a-Si with artificial graphite (Si:graphite = 5:95 in weight ratio). In the blend of c-Si and graphite (hereafter, c-SiC), crystalline peaks corresponding to both silicon and graphite are observed. However, in the XRD data of the mixture of a-Si and graphite (hereafter, a-SiC), only peaks corresponding to graphite are observed. Figure 2e,f depict SEM images of c-SiC and a-SiC, respectively. Although some areas exhibit slight silicon agglomerates, the overall dispersion of silicon on the surface of the artificial graphite in both blends appears to be satisfactory.

### 3.2. Electrochemical Performance

**Figure 3:**
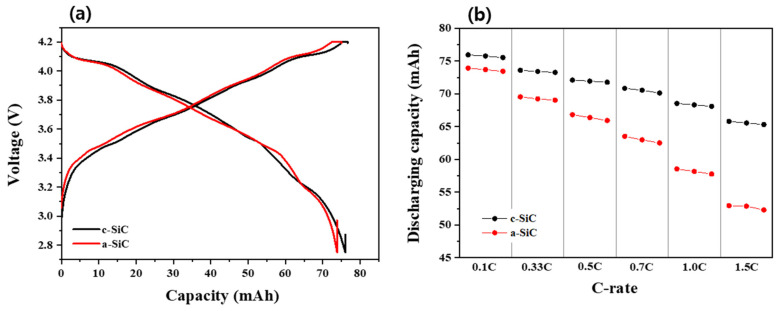
(**a**) Charge/discharge profiles at the first cycle. (**b**) Rate capability of c-SiC and a-SiC at incremental discharge rates from 0.1 C to 1.5 C. The charge rate is 0.1 C.

To compare the performance of c-SiC and a-SiC as anode materials in LIBs, double-layer pouch (DLP) cells (~80 mAh class) were assembled and evaluated. The design for the full cell is described in Table S1. The specific capacities of c-Si and a-Si (without mixing with graphite) were measured based on a half-coin cell, and are 3331.5 mAh/g and 1874.3 mAh/g in the range of 0.01 V to 1.5 V, respectively (Figure S1a). The anode materials employed in the DLP cells are blends of graphite and c-Si or a-Si in a weight ratio of 95:5, and their initial capacities are shown in Figure S1b. The capacities of c-SiC and a-SiC are 500.9 mAh/g and 393.8 mAh/g in the range of 0.01 V to 1.5 V, respectively. The initial charge–discharge profiles obtained after the formation process of the DLP cells are shown in Figure 3a, and their capacities are 75.9 mAh (c-SiC) and 73.7 mAh (a-SiC), respectively. The reason for presenting the full-cell characteristics is to demonstrate the practical ability of these materials using a “practical cell”, which incorporates all key components, including anodes, cathodes, and electrolytes, and possesses a capacity suitable for real-world applications, distinguishing it from coin cells used for preliminary material assessments.

As shown in Figure 3a, the charging voltage of a-SiC during initial charging is slightly higher than that of c-SiC. This phenomenon is thought to occur because the band gap of a-Si (1.8 eV) is higher than that of c-Si (1.1 eV) [58]. The C-rate properties were measured after initial charging/discharging, with a fixed charging rate of 0.1 C and varying discharging rates from 0.1 C to 1.5 C. As shown in Figure 3b, the C-rate capability of c-SiC is superior to that of a-SiC. This may be attributed to some of the silicon atoms of a-Si forming dangling bonds. As shown in Figure S2 in Supporting Information, the resistance of a-SiC obtained from the impedance spectrum was measured to be larger than that of c-SiC, which is consistent with the characteristics shown in the C-rate property and the initial charge/discharge voltage–capacity curve results.

**Figure 4:**
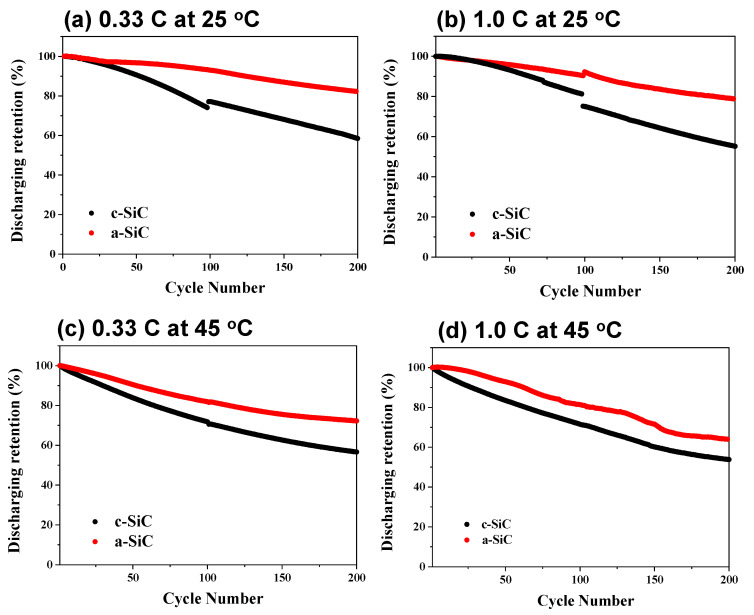
Cycling performance of discharge retention at (**a**) 0.33 C and 25 °C, (**b**) 1.0 C and 25 °C, (**c**) 0.33 C and 45 °C, and (**d**) 1.0 C and 45 °C. The charge rate of all measurements is 0.2 C.

Figure 4 depicts the cycle curves of DLP cells assembled with c-SiC and a-SiC under various conditions, with a-SiC showing significantly better cycle characteristics in all conditions. At a low temperature of 25 °C, a-SiC exhibits a capacity retention rate ~30% higher than c-SiC after 200 cycles at both low (0.2 C) and high (1.0 C) discharging rates. However, at a high temperature of 45 °C, the capacity retention of a-SiC is only ~20% higher than that of c-SiC at both discharging rates. There is a widely held opinion that the cycling ability of silicon anodes is closely related to mechanical and physical damage, such as the cracking of silicon particles due to the repeated expansion and contraction of silicon particles during charge and discharge [59,60]. In several studies from experiments and simulations, it is predicted that the extent of mechanical deformation in c-Si will exceed that in a-Si. This is attributed to the dependency of the lithiation reaction in c-Si on crystallographic planes, leading to anisotropic expansion and contraction, unlike isotropic lithiation in a-Si. In a study of the fracturing of c-Si nanopillars via electrochemical lithiation, Lee et al. suggested that the lithiation/delithiation of a-Si could lead to lower stresses or a different stress state than in c-Si, potentially making the fracture less ready in initially amorphous structures [41].

**Figure 5:**
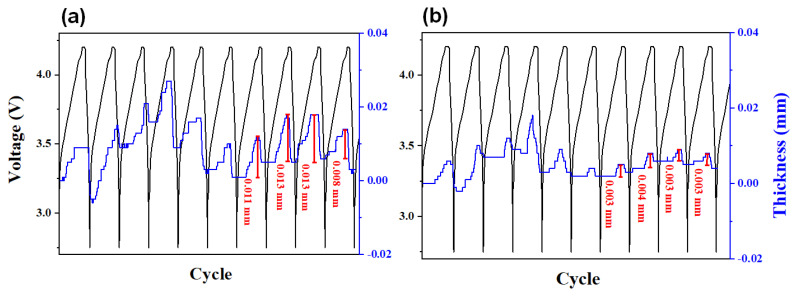
Voltage and thickness profiles of DLP (double-layer pouch) cells measured during initial 10 cycles: (**a**) c-SiC and (**b**) a-SiC.

In Figure 5, the thickness variation in DLP cells assembled with c-SiC and a-SiC was plotted as a function of cycling. In both cases, expansion during charging and contraction during discharging were observed repeatedly. After five cycles, the thickness variation in the c-SiC cell is significantly, ~three–four times, greater than that of the a-SiC cell. This indicates that the volume change in c-SiC is much larger than that of a-SiC. These results are consistent with the comparison of cycling properties between the two samples discussed earlier.

### 3.3. Silicon Nanofilm Volume Expansion Simulation

**Figure 6:**
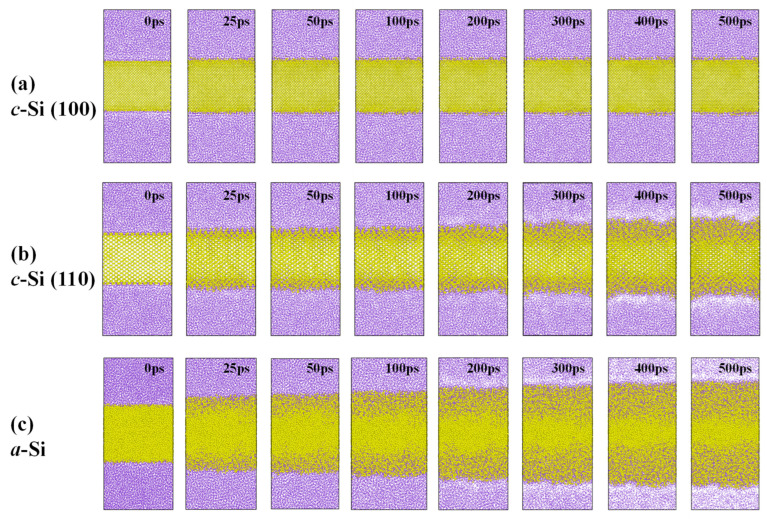
Snapshots of lithiation the process of (**a**) c-Si (100), (**b**) c-Si (110), and (**c**) a-Si nanofilms. (Purple: Lithium, Yellow: Silicon).

We describe the lithiation of silicon nanofilms with different entering planes (i.e., the interface between lithium and silicon) from the molecular dynamics simulation. As shown in Figure 6a,b, in c-Si, the reaction between silicon and lithium through the (100) plane is minor, while the penetration of lithium through the (110) plane of c-Si is clearly observed. This difference in lithiation behavior depending on the silicon crystal face into which lithium enters has been reported in other previous studies [61,62]. This phenomenon arises from the higher atomic density of c-Si along the [100] direction compared to the [110] direction. Consequently, penetrating through the [100] direction requires much higher activation energy. This characteristic is considered to underlie the specific direction preference in lithiation and the anisotropic expansion observed during lithiation in c-Si [39,41,63]. When lithium reacts with c-Si, it clearly forms an amorphized Li-Si alloy, as shown in Figure 6b, which has also been reported in a few studies looking at the electrochemical lithiation of c-Si [64,65]. Meanwhile, in the case of a-Si, as shown in Figure 6c, the extent of the reaction over ~50 ps is comparable to the reaction that occurred in c-Si through the [110] direction over ~400 ps, indicating that the lithiation of a-Si is much more facile compared to c-Si.

**Figure 7:**
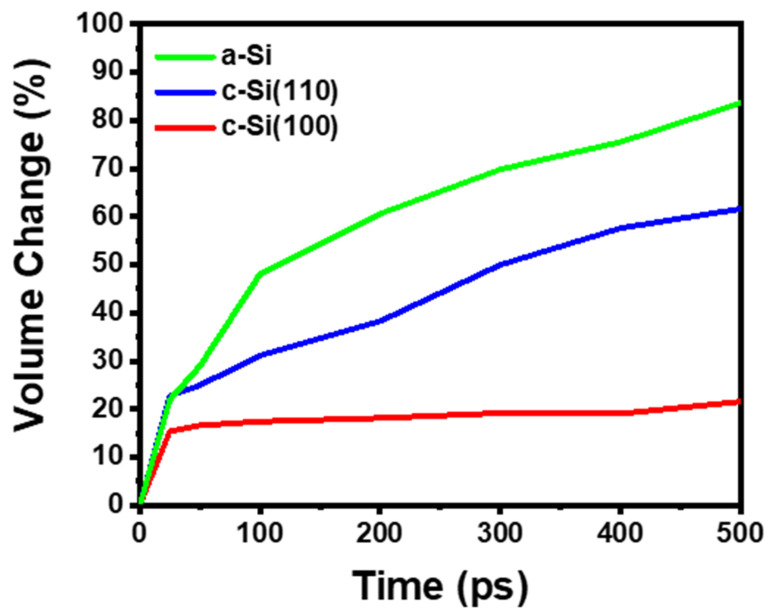
Volume change occurred during the lithiation of silicon nanofilms.

When Si undergoes lithiation, it experiences volume expansion. To quantify this, we assumed the distance between the silicon atoms furthest from the center of the film in both directions as the length of the edge in the z-direction and calculated the volume of a rectangular parallelepiped of LiSi alloy (Figure 7). In the lithiation of c-Si, lithium penetration in the [100] direction induces ~15% volume expansion during the initial 30 ps and then nearly saturates, with no further penetration being observed. However, in the [110] direction, after the initial rapid volume expansion, lithium continues to penetrate into the silicon film, resulting in ~50% volume expansion over 500 ps. On the other hand, in the lithiation of a-Si, the volume expansion over 500 ps reaches ~90%, indicating a significantly greater extent of lithiation compared to c-Si. There is some risk in directly correlating the volume expansion of silicon during lithiation in a molecular dynamics simulation and the thickness changes observed in the DLP cells during cycling in the previous experiment (Figure 5). This is because, from the perspective of lithiation kinetics in this simulation, a-Si lithiates much more readily, or rapidly, compared to c-Si. Therefore, before the maximum amount of lithium forms an alloy with silicon and reaches a stable state, we can expect that, when comparing the volume expansion due to lithiation over the same period, lithiation of a-Si will cause a greater volume expansion. In other words, if complete lithiation of both a-Si and c-Si systems occurs over a sufficient time, it could be speculated that the extent of volume expansion in the two systems might also be similar. We will address this point again in the later section where we discuss the experimental and atomistic modeling results in relation to each other.

The coordination number presented in Figure S3 provides a quantitative means to analyze the lithiation behavior of silicon. As shown in the volume change depicted in Figure 7, the rapid initial expansion and significant volume change tendency of a-Si align with the observed increase in the Si-Li coordination number. In contrast, as shown in Figure S3a,d, c-Si(100) exhibits a negligible volume change, corresponding to minimal variations in both the Si-Si and Si-Li coordination numbers. However, in c-Si(110) and a-Si, where the volume change is pronounced, the Si-Si coordination number shows a decreasing trend and the Si-Li coordination number increases. Notably, in a-Si, where the volume change is more substantial (~90%), the four-fold coordinated Si-Si bonds decrease significantly and the fraction silicon atoms with two- and three-fold coordination rise as the simulation progresses. This trend, highlighted by the increasing Si-Li coordination number in Figure S3f, elucidates the large volume expansion caused by the rapid lithiation of a-Si.

**Figure 8:**
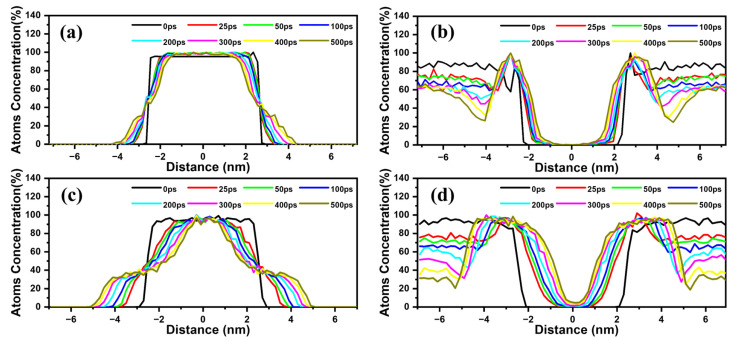
Variation in density profiles during the lithiation process: (**a**) Si and (**b**) Li in c-Si (110) nanofilm, (**c**) Si and (**d**) Li in a-Si nanofilm.

The concentration profiles depicted in Figure 8 also illustrate the extent of lithiation of the silicon nanofilms. Initially, the profiles of lithium and silicon exhibit a very steep gradient between the lithium reservoir and the silicon film in both cases. As time progresses, they display a similar pattern where the slope gradually decreases, suggesting that both c-Si and a-Si are undergoing lithiation. However, it is evident that the lithiation rate of a-Si is significantly faster than that of c-Si. This can be attributed to the lower activation barrier and faster lithium diffusion in a-Si compared to c-Si. This observation aligns with a study of Tritsaris et al., which reported a lower energy barrier for lithium diffusion in a-Si (<0.50 eV) compared to c-Si (0.55 eV) [66].

### 3.4. Effects of Bond Strength and Stress

**Figure 9:**
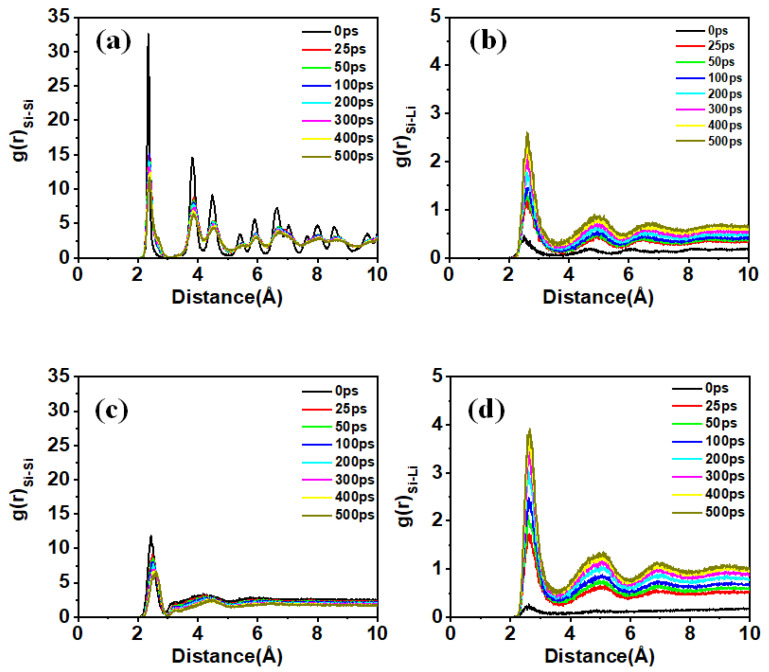
Variation in radial distribution functions during lithiation process: (**a**) Si-Si and (**b**) Si-Li in c-Si (110) nanofilm, (**c**) Si-Si and (**d**) Si-Li in a-Si nanofilm.

The time-resolved radial distribution functions (RDFs) show the evolution of the internal microstructure of silicon nanofilms during lithiation (Figure 9). Both c-Si and a-Si nanofilms exhibit a significant decrease in the height of peaks of the Si-Si RDFs, along with a slight shift to larger distances. This indicates that the strength of Si-Si bonds is gradually weakening and that the Si atoms around the Si atoms are being replaced by Li atoms. In the case of c-Si, it is observed that the crystallinity is maintained to some extent even after 500 ps. Furthermore, the Si-Li RDFs of the two systems exhibit quite similar patterns of change. However, in the case of a-Si, the increase in intensity of the first peak occurs significantly faster than in c-Si, indicating that the lithiation process in a-Si is much faster. Overall, the comparative analysis of the lithiation phenomena between c-Si and a-Si nanofilms is consistent with Chen et al.’s study [61].

**Figure 10:**
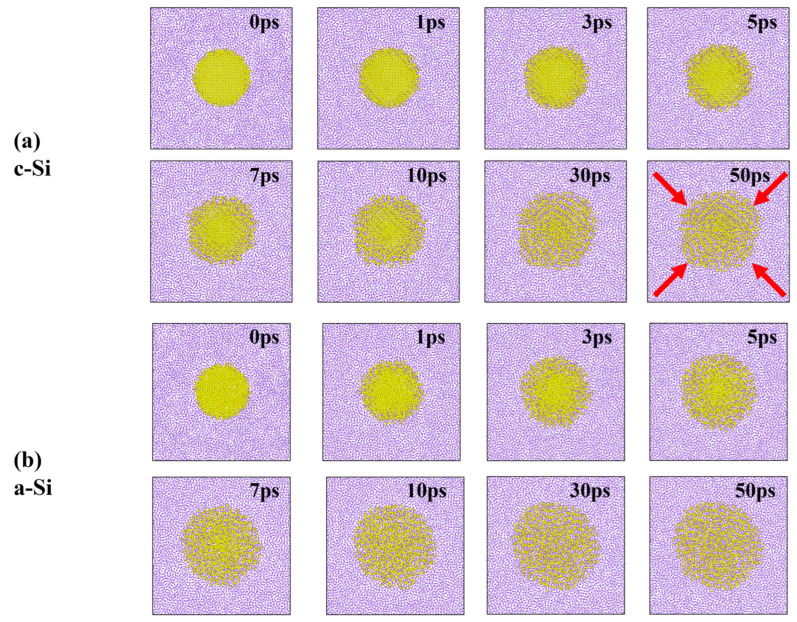
Snapshots of the lithiation process: (**a**) c-Si and (**b**) a-Si spherical nanoparticles. The red arrows in the 50 ps snapshot of (**a**) indicate the (110) planes.

Then, what would the lithiation phenomenon be like in spherical nanoparticles? As expected, it appears to occur more rapidly in a-Si than in c-Si (Figure 10). After 10 ps, in the former, lithium atoms have penetrated to the center of the silicon, while, in the latter, it appears that there is still a region of pure silicon remaining around the center. Additionally, even after 50 ps, crystalline traces are still visible in c-Si. Meanwhile, in a-Si, lithiation progresses uniformly from all radial directions. In contrast, in c-Si, lithiation seems to be more prominent in an anisotropic fashion along the [110] direction, as indicated by the red arrows. The time-dependent RDFs of the nanoparticles are quite similar to those of the nanofilms (Figure S2 in Supporting Information).

**Figure 11:**
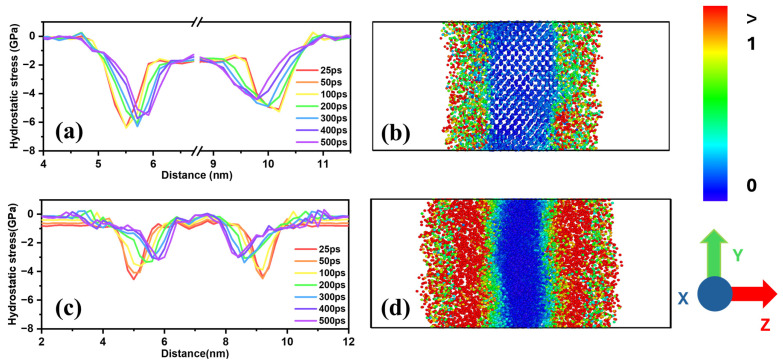
(**a**) Variation in stress profile in the z-direction and (**b**) atomic strain distribution after 500 ps during lithiation of the (110) plane of c-Si. (**c**) Variation in stress profile in the z-direction and (**d**) atomic strain distribution after 500 ps during lithiation of a-Si.

It is very difficult to experimentally measure the mechanical stress generated during the lithiation process caused by electrochemical reactions. However, it is possible to numerically calculate this stress through atomistic simulations. When lithium atoms diffuse into silicon, they squeeze between the silicon lattices, causing the lattice spacing to expand, increasing the volume and resulting in the accumulation of stress. As lithium atoms penetrate into silicon, they alter the interactions with silicon atoms, leading to changes in the distances and bonding strengths between surrounding atoms [67]. These changes induce significant stress in the silicon lattice and also affect the diffusion rate of lithium atoms. Figure 11a,b show the stress variations over time and the atomic strain distribution after 500 ps, respectively, when lithium penetrates the (100) plane of c-Si, while Figure 11c,d present the results for a-Si during lithiation.

Comparing Figure 11a,c, a considerable stress of approximately −6 GPa initially forms at the lithium–silicon interface for c-Si, whereas it is relatively small at about −4 GPa for a-Si. The maximum negative stress location shifts more prominently in c-Si than in a-Si. As the simulation progresses, the stress interface in a-Si rapidly moves toward silicon, indicating deep lithium penetration due to the rapid lithiation of a-Si. Additionally, compressive stress is lower in a-Si. The inverse relationship between compressive stress and strain confirms that a-Si, exhibiting high strain, experiences lower compressive stress. This suggests that compressive stress at the interface delays lithiation at the boundary.

**Figure 12:**
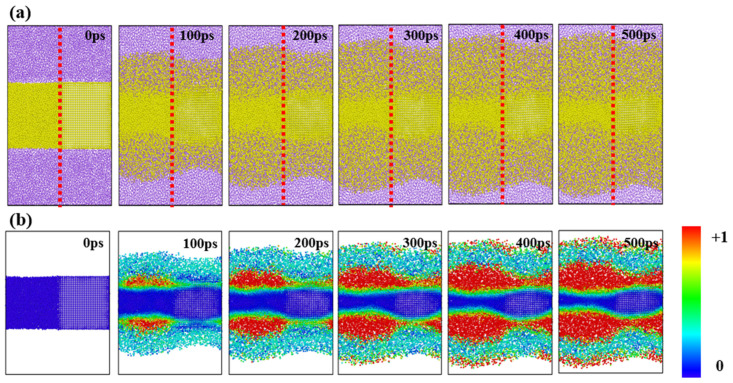
(**a**) Snapshots of the lithiation process and (**b**) atomic strain distribution in crystalline-amorphous combined nanofilms. The red dotted line divides the regions: (Left) a-Si, (Right) c-Si (110).

The cross-sectional strain distribution shown in Figure 11b,d also supports this explanation. In the case of a-Si, the extent of deformation is greater, and the region where deformation occurs is broader compared to c-Si. This aligns well with a previous study, which has shown that, in amorphous silicon, phase changes are more flexible, leading to greater stress relaxation compared to c-Si [68]. By creating a nanofilm combining amorphous and crystalline silicon and observing the lithiation reaction, the behavior of both systems can be compared simultaneously. Figure 12 shows snapshots of the lithiation process over time and the atomic strain distribution for the crystalline–amorphous combined nanofilms. As shown in the previous results, deformation and lithiation occur more rapidly in the amorphous region compared to the crystalline region.

### 3.5. Comparison of DLP Cell and Simulation

In the experimental results discussed in the early section, the change in volume due to lithiation and delithiation of c-SiC was much greater than that of a-SiC. On the contrary, at first glance, the degree of volume change estimated through computer modeling appears larger for a-Si compared to c-Si. However, this is because a-Si lithiation occurs more rapidly than with its counterpart. In other words, when comparing the extent of change over the same period, the volume change in a-Si is greater than that of c-Si. The degree of volume changes in both systems with respect to the extent of lithiation (i.e., the amount of lithium penetrating) is nearly the same. Nevertheless, it might seem that the modeling results do not fully explain the volume changes observed in the experiments. Our explanation for this is as follows: as shown in the modeling results of this study, the initial stress at the lithium–silicon interface is greater in c-Si than in a-Si. In the case of a-Si, this stress is significantly alleviated as lithiation progresses; however, in c-Si, it is not, and, ultimately, the accumulated stress may lead to fracturing. Although this can vary depending on various conditions, such as the rate of lithiation, the study by Lee et al. suggests that, for c-Si, a fracture can only occur when the particle size exceeds a few hundred nm, while the critical size for a-Si is even larger [41]. Most molecular modeling studies, however, cannot directly observe this phenomenon due to limitations in time and the size of systems. Moreover, the c-Si we handled in our experiments is a polycrystal composed of several crystal planes joined together. When the anisotropic expansion and cracking of many anisotropic crystal grains are combined, it is expected to result in much larger macroscopic changes compared to the deformation observed in a-Si. Unfortunately, our study cannot address such macroscopic deformations. However, by comparing the changes in stress due to lithiation, we present indirect evidence for this explanation.

Meanwhile, including this work, when reviewing studies on silicon lithiation through molecular dynamics simulations, a recurring question arises. When we extend the time and length scales of lithium penetration into silicon obtained from modeling to the charging and discharging of experimentally observable anode materials, the actual charge and discharge process is several orders slower than what is estimated through computer modeling. How can we explain this discrepancy between experiments and simulations? Several points discussed in a previous study provide a rather convincing explanation for this issue [52]. First, unlike the defect-free modeling system for computer simulation, the actual electrode materials used in experiments contain significant defects. These defects increase the activation energy for lithium ion movement, lowering lithium mobility, which becomes one of the factors contributing to the gap between experimental and modeling results. In addition, in a real battery cell, beyond lithium and electrode materials, there are other components that influence lithium movement, such as electrolytes, binders, and separators. Thus, the bottleneck for experimental lithium ion mobility may not necessarily be the interdiffusion between atoms in the electrode materials and lithium atom. Therefore, without a clear understanding of these factors, we cannot conclude that the molecular modeling results, which appear to be significantly different from the experimental outcomes, are inherently limited. Hence, the diffusion coefficients of lithium ions obtained from this study or other computational simulation studies are highly meaningful in that they present an upper bound for the kinetic performance, such as the rate characteristics, of batteries.

## 4. Conclusions

This study directly compares the suitability of c-Si and a-Si of similar size by examining various properties of composite materials, each mixed with graphite, designed for use as anode materials in LIBs. The “bulk” silicon particles used in this work offer an advantage in terms of cost-effectiveness for actual product manufacturing as they have not undergone secondary processing, such as the introduction of costly nanostructures. It is well known that c-Si has a greater capacity per unit mass compared to its amorphous counterpart. However, due to its irreversible structural vulnerability through repeated charging and discharging, its practical industrial application is less effective than that of a-Si. The a-Si/graphite composite exhibited relatively better performance in terms of both rate and temperature characteristics. This is believed to be related to the volume changes during charging and discharging arising from stress differences at the Si-Li interface. Despite the inherent limitations of a molecular dynamics simulation in handling time and length scales that can be directly compared to real experiments, the modeling results obtained in this study offer several insights. The results showed that the activation energy for lithium migration in a-Si is lower than that in c-Si, resulting in a higher degree of lithiation within the same time frame. This suggests that, in c-Si, lithiation induces great stress, which could lead to more severe mechanical failure on a macroscopic scale. In addition, as seen in the modeling results of this study, unlike a-Si, c-Si exhibits anisotropic lithium penetration. Therefore, in the case of polycrystalline silicon, where multiple crystal planes grow together, expansion and the resulting fractures will also occur anisotropically. As a result, c-Si will experience more severe volume expansion compared to a-Si, which aligns well with our experimental results. Consequently, this study provides guidelines for designing silicon anodes for practical LIBs for the next generation, emphasizing the need to balance the high capacity of c-Si with the superior cyclability of a-Si. This study aimed to investigate lithiation behavior using a molecular dynamics simulation which focused on atomic-level movements. Due to the limitations of the simulation, it was not possible to replicate the actual cell scale, and several constraints were identified. Simulation approaches at the bulk level, such as the finite element method (FEM), can address these limitations [69]. Future studies should focus on optimizing the simulation methods to reduce costs and further investigating the effects of graphite and silicon content on their performance. This will contribute to the development of graphite–silicon hybrid anode materials.

## Figures and Tables

**Table 1:** Specifications of nanofilms and nanospheres for molecular dynamics simulations.

System	Nanofilm			Nanosphere	
	c-Si (100)	c-Si (110)	a-Si	c-Si	a-Si
Cell size (Å^3^)	70 × 70 × 160	70 × 70 × 155	70 × 70 × 140	130 × 130 × 130	130 × 130 × 130
Number of Si atoms	12,506	12,506	12,145	3223	3456
Number of Li atoms	20,630	20,630	21,883	97,013	97,013

## Data Availability

The original contributions presented in this study are included in the article and Supplementary Materials. Further inquiries can be directed to the corresponding authors.

## Supplementary Materials

The following supporting information can be downloaded at https://www.mdpi.com/article/10.3390/ma18030515/s1, Table S1: Detailed specifications of the pouch-type full cells; Figure S1: Initial charge–discharge curves of the half-coin cells made with the Si materials used in the DLP cells; Figure S2: Nyquist plots from EIS measurements; Figure S3: Variations in the coordination number of Si-Si and Si-Li obtained from molecular dynamics for lithiation of c-Si and a-Si nanofilms; Figure S4: Distribution of the Si-Si bond length of the initial state of c-Si and a-Si nanofilms; Figure S5: Variations in the radial distribution functions obtained from the molecular dynamics for the lithiation of c-Si and a-Si nanospheres.
