# Supplementary material for: Comparison Between Crystalline and Amorphous Silicon as Anodes for Lithium Ion Batteries: Electrochemical Performance from Practical Cells and Lithiation Behavior from Molecular Dynamics Simulations
Source: Materials (Basel). 2025 Jan 23;18(3):515. doi: 10.3390/ma18030515 (PMC11818356; doi:10.3390/ma18030515)
Supplement: Supplementary file 1 [file materials-18-00515-s001.zip › materials-3404284-supplementary.pdf]

<Supporting Information>

Comparison Between Crystalline and Amorphous Silicon as Anodes for Lithium Ion Batteries: Electrochemical Performance from Practical Cells and Lithiation Behavior from Molecular Dynamics Simulations

Geonhee Kim [1], Min-Ji Yang [2], Sanghun Lee [1,*] and Jae-Hyun Shim [2,*]

[1] Department of Chemistry, Gachon University, Seongnam 13120, Republic of Korea; geonheekim@gachon.ac.kr
[2] Department of Energy System Engineering, Dongshin University, Naju 58245, Republic of Korea; g6847@naver.com
* Correspondence: sanghunlee@gachon.ac.kr (S.L.); jhshim@dsu.ac.kr (J.-H.S.)

Table S1. Schematic Diagram of the DLP cell (top left), a photo of the assembled DLP cell (top right), and its detailed specifications.

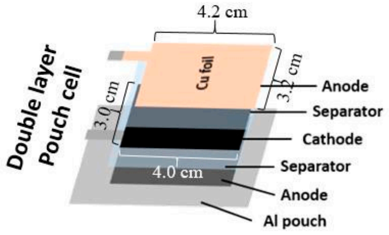

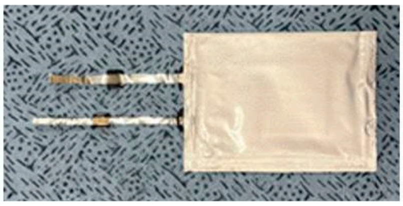

Double Layer Pouch cell	Electrode	Electrode Capacity (mAh)	Area(cm^2)	Loading Level (g/cm^2)	Materials capacity (mAh/g)	Tap density (g/cc)	N/P ratio
c-SiC	cathode	80.96	24	0.017	205	3.4	1.10
	anode	89.18	26.88	0.0069	500.9	1.5	
a-SiC	cathode	80.96	24	0.017	205	3.4	1.10
	anode	89.42	26.88	0.0088	393.8	1.5	

The cathode active material applied to the (+) electrode was LiNi0.8Co0.1Mn0.1O2 (NCM811). The conductive agents used were carbon black (Cabot) and multi-walled carbon nanotubes (CNT, Cabot) mixed in a ratio of 0.36:0.84 wt%. The binder was polyvinylidene fluoride (Pvdf), dispersed in NMP. The cathode slurry, with a total solid content of 65 wt%, was mixed using a P/D mixer with a 700 g base.

After evaluating the cathode capacity using the coin half-cell (cathode capacity, 205 mAh/g), it was applied to the DLP cell design. The loading level of the one-sided electrode was set at 0.017 g/cm^2, with a tap density of 3.5 g/cc, a double-sided electrode thickness of 100 μm, an electrode area of 3.0 × 4.0 cm, and a cell design based on a discharge capacity of 80.96 mAh. The coated cathode electrode was dried in an oven at 120°C and subjected to vacuum drying (V.D.) to ensure drying. Rolling was used to maintain a composite density of 3.5 g/cc.

The anode active materials for the (-) electrode were a mixture of artificial graphite, c-Si, or a-Si in a ratio of 95:5, respectively. The conductive agents were carbon black (C.B.) and single-walled carbon nanotubes (CNT, Tuball) mixed in a ratio of 0.9:0.1 wt%. The binder was a water-based mixture of CMC (carboxymethyl cellulose) and SBR (styrene butadiene rubber) in a 1:2 wt% ratio. The anode slurry, with a total solid content of 50 wt%, was mixed using a P/D mixer with a 600 g base.

After evaluating the cathode capacity using the coin half-cell (the capacities of c-SiC and a- SiC were 500.9 mAh/g and 393.8 mAh/g, respectively), it was applied to the DPL cell design. Considering the anode electrode capacity of ~89 mAh (discharge capacity basis) and ensuring an N/P ratio of 1.10, the design was based on the cathode electrode capacity of 80.96 mAh (discharge capacity basis). The c-SiC anode electrode was coated to maintain a one-sided electrode loading level of 0.0069 g/cm^2 with a thickness of 46 μm. The a-Si anode electrode was coated to maintain a one-sided electrode loading level of 0.0088 g/cm^2 with a cross-sectional electrode thickness of 58 μm. Both electrodes had the same tap density of 1.5 g/cc. At this time, the anode electrodes with c-SiC and a-SiC materials were both designed with the same size of 3.2 × 4.2 cm and a discharge capacity of approximately 89 mAh. The fabricated anode electrodes were dried in an oven at 100°C and subjected to vacuum drying (V.D.), with the tap density being maintained at 1.5 g/cc through rolling. All processes after coating were conducted in a dry room. The design model and design table of the DLP cell are shown in Table S1.

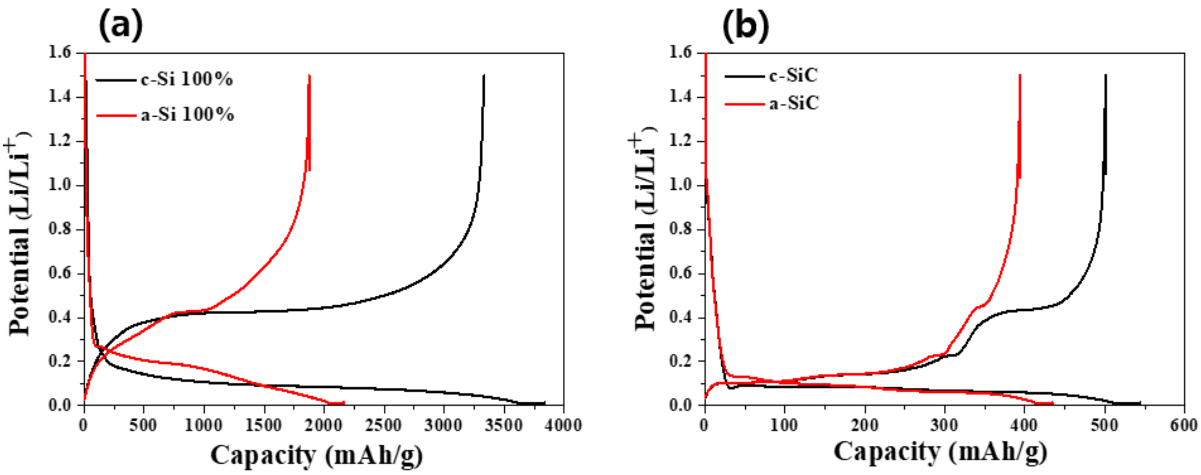

Figure S1. Initial charge–discharge curves of the half-coin cells made with the Si materials used in the DLP cells: (**a**) charge–discharge curves of c-Si and a-Si (without graphite), (**b**) those of c-SiC and a-SiC.

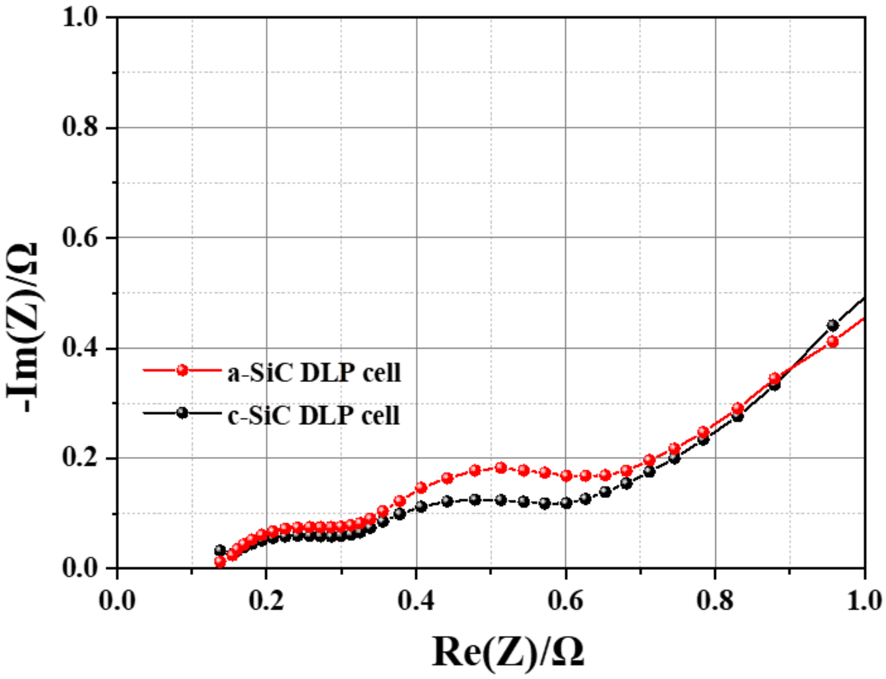

Figure S2. Nyquist plots of *c*-SiC and *a*-SiC DLP cells obtained from EIS spectroscopy.

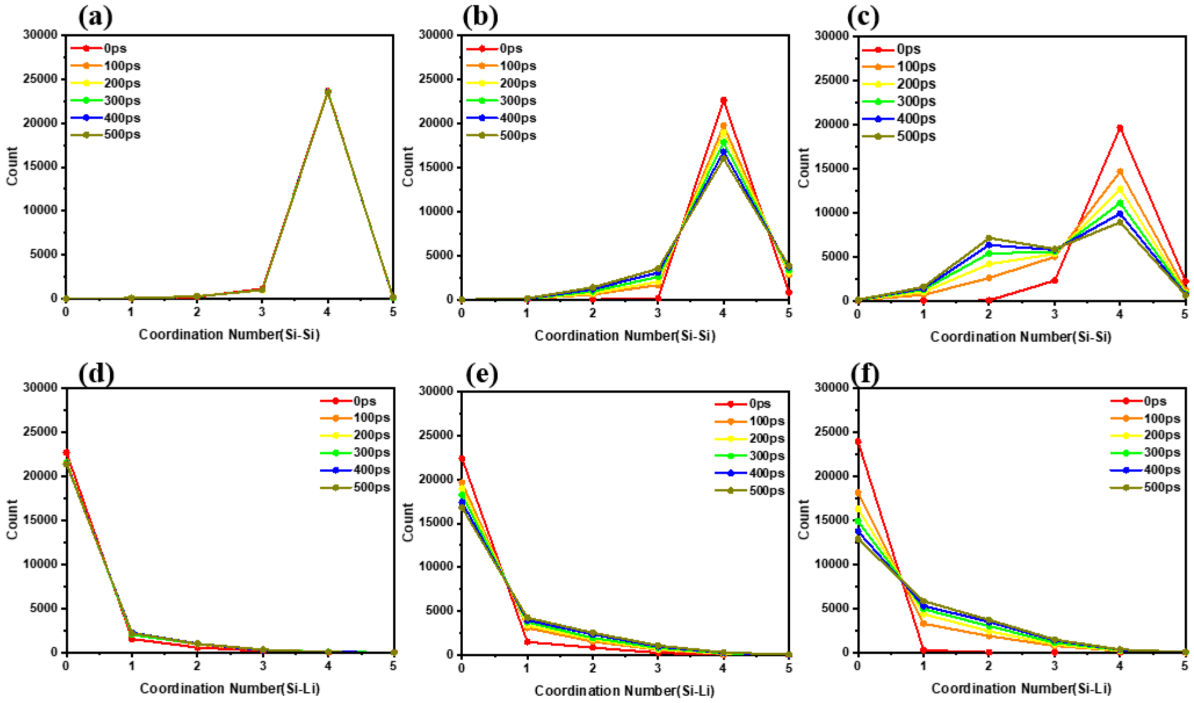

Figure S3. Variation in coordination number of Si-Si for (a) *c*-Si (100), (b) *c*-Si (110), and (c) *a*-Si; Si-Li for (d) *c*-Si (100), (e) *c*-Si (110), and (f) *a*-Si nanofilms during lithiation.

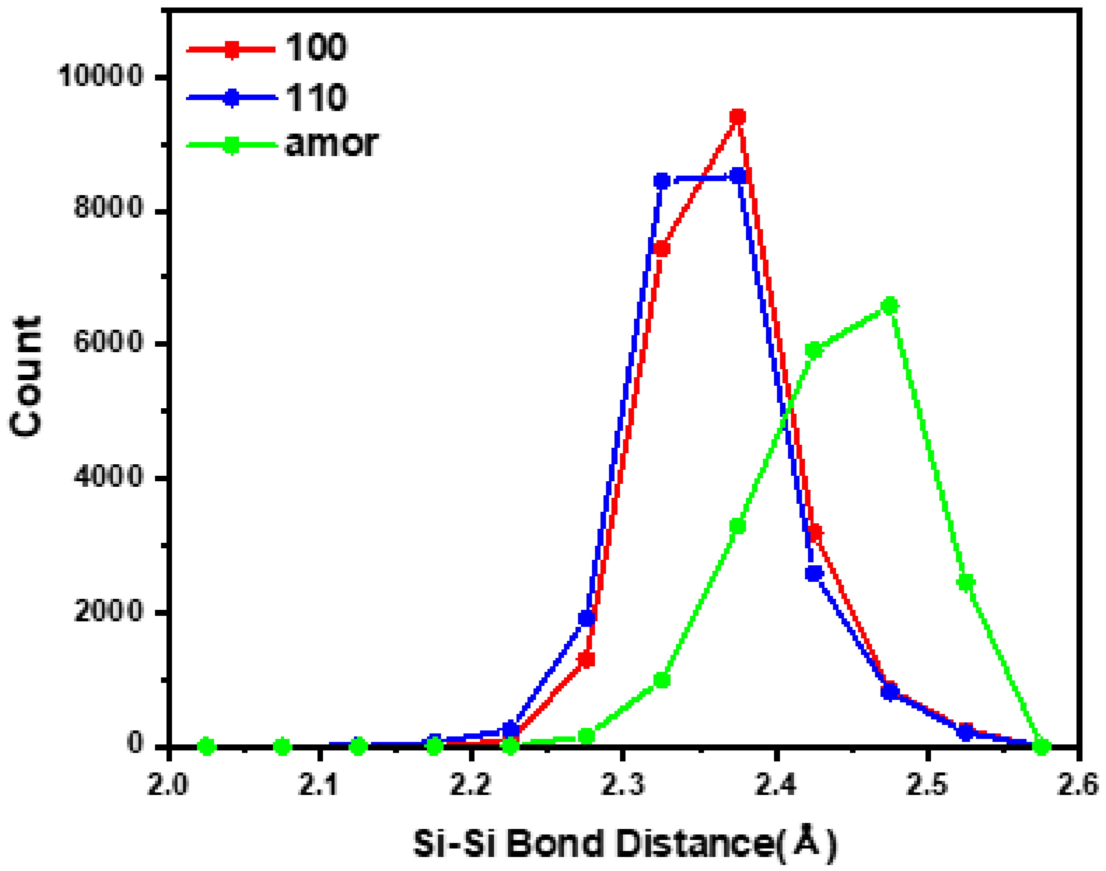

Figure S4. Distribution of the Si-Si bond length in the initial state of silicon nanofilms.

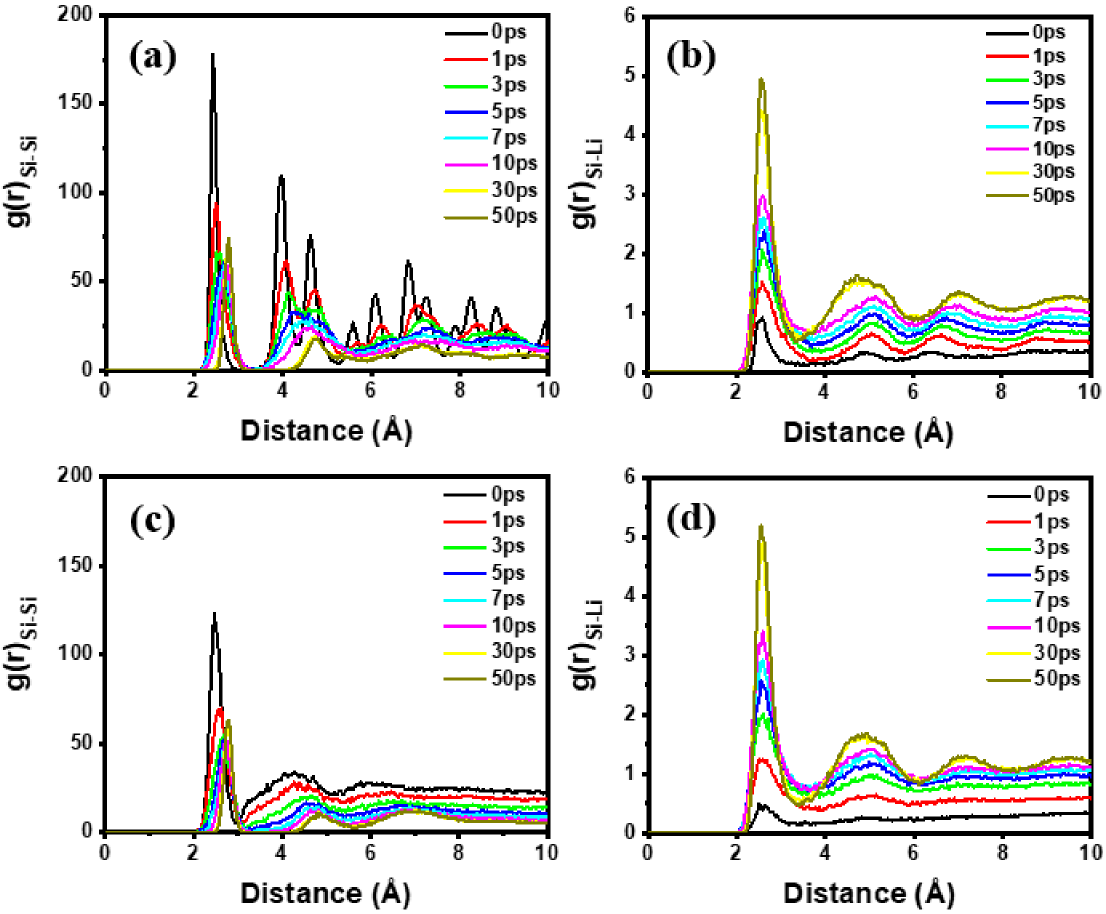

Figure S5. Variation in radial distribution functions during the lithiation process: (a) Si-Si and (b) Si-Li in *c*-Si nanosphere, (c) Si-Si and (d) Si-Li in *a*-Si nanosphere.
